# Roles of DNA damage in renal tubular epithelial cells injury

**DOI:** 10.3389/fphys.2023.1162546

**Published:** 2023-04-06

**Authors:** Peipei Wang, Jing Ouyang, Zhanjun Jia, Aihua Zhang, Yunwen Yang

**Affiliations:** ^1^ Department of Nephrology, Children’s Hospital of Nanjing Medical University, Nanjing, China; ^2^ Nanjing Key Laboratory of Pediatrics, Children’s Hospital of Nanjing Medical University, Nanjing, China; ^3^ Jiangsu Key Laboratory of Pediatrics, Nanjing Medical University, Nanjing, China

**Keywords:** DNA damage, tubular epithelial cells, DNA repair, DDR, AKI (acute kidney injury), CKD—chronic kidney disease

## Abstract

The prevalence of renal diseases including acute kidney injury (AKI) and chronic kidney disease (CKD) is increasing worldwide. However, the pathogenesis of most renal diseases is still unclear and effective treatments are still lacking. DNA damage and the related DNA damage response (DDR) have been confirmed as common pathogenesis of acute kidney injury and chronic kidney disease. Reactive oxygen species (ROS) induced DNA damage is one of the most common types of DNA damage involved in the pathogenesis of acute kidney injury and chronic kidney disease. In recent years, several developments have been made in the field of DNA damage. Herein, we review the roles and developments of DNA damage and DNA damage response in renal tubular epithelial cell injury in acute kidney injury and chronic kidney disease. In this review, we conclude that focusing on DNA damage and DNA damage response may provide valuable diagnostic biomarkers and treatment strategies for renal diseases including acute kidney injury and chronic kidney disease.

## 1 DNA damage in general

DNA damage is an alteration in DNA’s physical or chemical structure that produces a modified DNA molecule, that is distinct from what originally existed (Chatterjee and Walker, 2017). DNA damage arises from environmental variables and common cellular metabolic processes, resulting in damage of DNA structure and impairing the DNA replication mechanism (Parker et al., 2017). DNA damage occurs and brings about 10^4^ ∼ 10^6^ molecular lesions in each cell every day.

**FIGURE 1:**
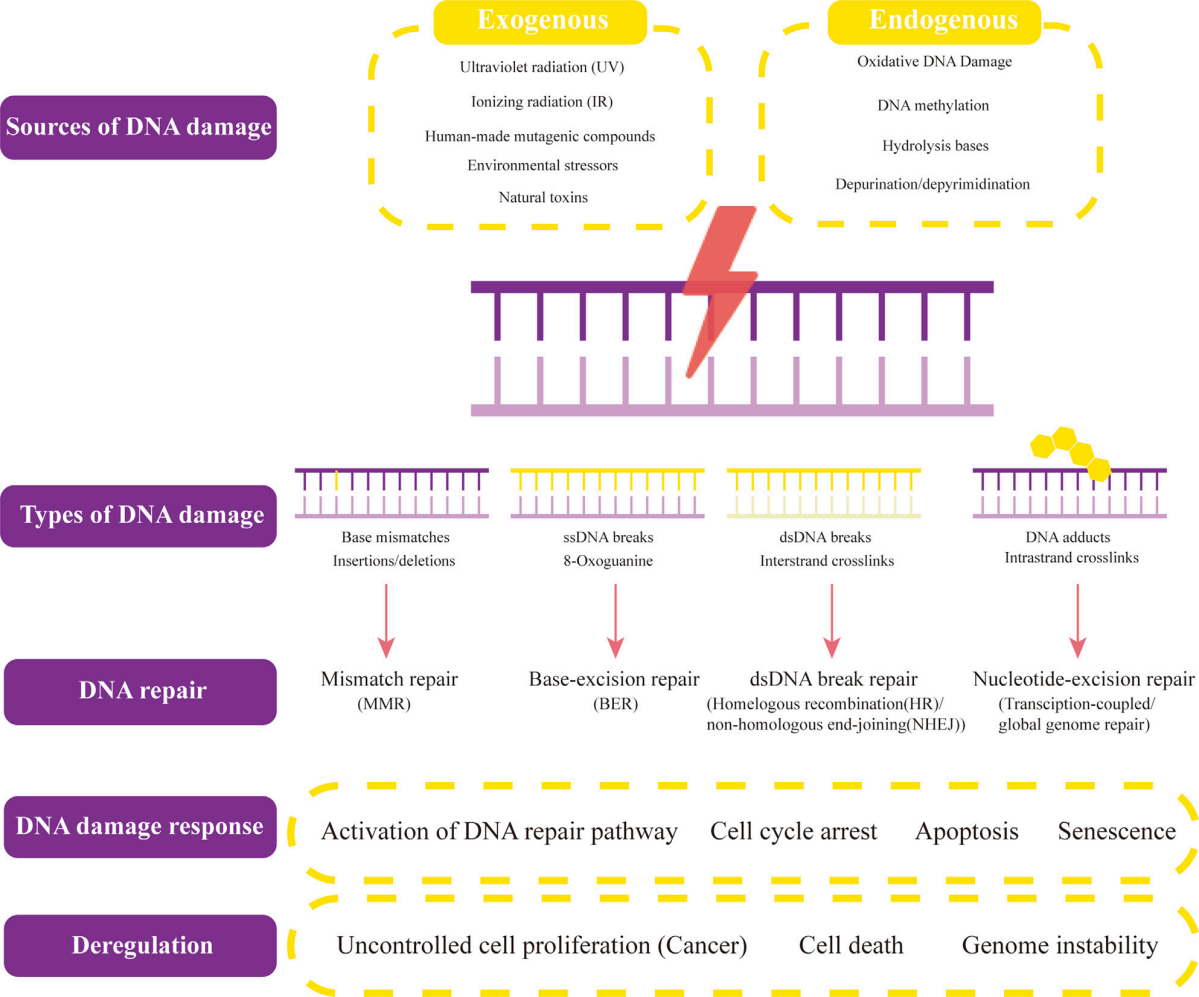
Sources and types of DNA damage and DNA repair. Both exogenous factors and endogenous factors can cause DNA damage. Exogenous factors contain ultraviolet radiation, ionizing radiation, human-made mutageniccompounds, environmental stressors, and other natural toxins. Endogenous biological activities can damage DNA in many ways, including oxidative DNA Damage, DNA methylation, hydrolysis of bases, and depurination/depyrimidination. The common types of DNA damage include base mismatches (insertions/deletions), ssDNA breaks (8-Oxoguanine), dsDNA breaks (Interstrand crosslinks), and DNA adducts (intrastrand crosslinks). Their corresponding DNA repair methods are mismatch repair (MMR), base-excision repair (BER)/non-homologous end-joining (NHEJ), and transcription-coupled/global genome repair. When different types of DNA lesions activate DNA-damage response proteins, DNA repair begins. The signaling pathways that these proteins then activate include those for DNA repair, cell cycle arrest, apoptosis, and senescence. Uncontrolled cell proliferation (cancers), cell death, and genome instability result from dysregulation of DNA damage response and repair, and several of these proteins offer potential therapeutic targets.

In human cells, both exogenous factors from nature or environment and endogenous factors from metabolic activities in cellular can cause DNA damage. Exogenous damage can be caused by external agents such as ultraviolet [UV 200–400 nm] radiation (Liu et al., 2015) from the Sun or other artificial light sources, other ionizing radiation including x-rays and gamma rays (Jackson and Bartek, 2009), hydrolysis or thermal disruption, certain plant toxins, humanmade mutagenic chemicals especially aromatic compounds that act as DNA intercalating agents and viruses (Chatterjee et al., 2016, Chatterjee et al., 2015), Endogenous biological activities can damage DNA in many ways, including oxidative DNA Damage (Cooke et al., 2003), DNA methylation (Ehrlich, 2009), hydrolysis of bases [such as spontaneous base deamination and depurination/depyrimidination (Yonekura et al., 2009)], mismatch of bases (which is caused by mistakes in DNA replication, which sew the incorrect DNA base into a newly formed DNA strand or accidentally skip over or insert a DNA base), the formation of the adduct [including monoadduct, diadduct, and bulky adduct (Lindahl and Barnes, 2000)]. DNA methylation along with histone modification, chromatin rearrangement, and variations in non-coding RNA transcript levels is one method of epigenetic control. Numerous studies have demonstrated that it is crucial for AKI, the transition from AKI to CKD, and renal repair (Guo et al., 2019) (Figure 1).

Physiological processes such as aerobic metabolism, lysosome biogenesis, peroxisome interactions, or the second phase of metabolism could result in the formation of reactive oxygen species (ROS) and Reactive Nitrogen Species (RNS). Being extremely reactive, the most common ROS that damages DNA is the hydroxyl radical (Son et al., 2008). ROS can induce single or double-strand breakage, nucleotide alterations, deoxyribose changes, and DNA cross-linking in DNA. If oxidative DNA damage is not repaired before DNA replication, it could lead to cell death, DNA mutation, replication mistakes, and genomic instability (Cooke et al., 2003; Schupp et al., 2016).

DNA repairing is a cellular response to DNA damage that may restore the DNA structure and function; however, sometimes it does not eliminate the DNA damage, but only enables the cell to tolerate the DNA damage and keep survive (Jeggo et al., 2016). If the damage is not fully repaired, the remains will show up under the right conditions, but if the cell without DNA repair, it will not be able to cope with the frequent DNA damage events and will not survive (Jackson and Bartek, 2009).

Although most DNA damage can be repaired, such repair is not always effective. Unrepaired DNA damage builds up in non-replicating cells, such as those in adult mammals’ brains or muscles, which could lead to aging and cancer (Ciccia and Elledge, 2010; Soria-Valles et al., 2017; Al Zouabi and Bardin, 2020; Patel et al., 2020). Cells such as the colon cells will make mistakes during replication when the damage occurs in the DNA template strand. These mistakes may result in mutations or epigenetic changes (Freitas and de Magalhaes, 2011). Both variations are replicable and transferable to succeeding cell generations. These modifications may alter how genes work or how gene expression is regulated, which may speed up the development of cancer.

Additionally, some evidences point out that DNA damage may play an importmant role in renal diseases. This review will elucidate DNA damage, its types and location, and its pathological role in renal tubular epithelial cells (TECs) in AKI and CKD caused by different factors. Recent advances in DNA repair and DDR are also reviewed. It might be useful in identifying AKI and CKD early signs. It will also emphasize renoprotective techniques by focus on DNA damage to lessen kidney damage from various factors.

## 2 Nucleus DNA and mitochondria DNA

DNA can be found in the mitochondria as well as the nucleus of human cells and other eukaryotic cells. The mitochondria contain several copies of mitochondrial DNA (mtDNA). Additionally, it forms the nucleoid complex by being firmly linked to several proteins. Oxidative phosphorylation (OXPHOS) in mitochondria produces large amounts of Adenosine triphosphate (ATP) which is the main source of energy for life activities. Many of the proteins to form the five OXPHOS complexes are encoded in nuclear DNA (nDNA), and a tiny subset of OXPHOS-related genes is encoded by mtDNA. ROS and free radical production in the mitochondria are byproducts of OXPHOS, and the highly oxidative environment these two byproducts produce damages the DNA in the mitochondria. The most frequently addressed pathological damage, ROS-induced damage, can result in mitochondrial DNA damage. There are other factors connected to DNA damage than oxidative damage. Some mtDNA damage may be linked to greater ROS levels, whereas others may not (Mattiazzi et al., 2004). There have been reports of low amounts of mtDNA damage in the *postmortem* brains of old patients or patients with neurodegenerative disorders, but these low levels have not been directly linked to higher ROS (Coppede and Migliore, 2015; Nissanka and Moraes, 2018). Nuclear or mitochondrial DNA damage, that is not repaired or that is incorrectly repaired results in cell cycle arrest, apoptosis, or mutations and can have deadly repercussions including premature aging (Herbert et al., 2008; Li et al., 2008), vascular disease (Mercer et al., 2007; Borghini et al., 2013), or cancer (Poulsen et al., 1998; Hoeijmakers, 2007).

## 3 DNA damage in renal diseases

### 3.1 Cisplatin-induced AKI (nephrotoxin)

Researchers first learned that platinum compounds could prevent cell division in 1960s. The U.S. Food and Drug Administration granted cisplatin approval in treating testicular and ovarian cancer in 1978 (Basu and Krishnamurthy, 2010). Now, Cisplatin is still widely used to treat a variety of solid tumors including bladder cancer, head and neck cancer, esophageal cancer, and so on. Despite being an effective chemotherapeutic drug in the clinic more than 40 years, the main adverse effect of cisplatin, nephrotoxicity, still limited its use as a safe anticancer drug (Manohar and Leung, 2018). Renal protection measures currently being applied include 1) extensive hydration in chemotherapy patients required to clear cisplatin from the kidneys and 2) the use of other platinum derivatives with fewer side effects such as carboplatin and oxaliplatin, but the therapeutic range of such drugs is also relatively narrow (Kruger et al., 2015). Despite these protective measures, the morbidity of AKI occurring in chemotherapy patients receiving cisplatin is still very high (Dasari and Tchounwou, 2014), AKI was found in 85/124 patients (69%) of those with locally advanced squamous cell carcinoma of the head and neck (LA-SCCHN) who were receiving cisplatin treatment, according to a retrospective cohort study assessing the incidence of AKI (McMahon et al., 2018). There is growing evidence that DNA damage and associated DDR play a key role in cisplatin-induced AKI (Basu and Krishnamurthy, 2010; Tchounwou et al., 2021).

Tubular epithelial cells (TECs) is one of the most damaged cells in cisplatin induced AKI (Xu et al., 2015). The well-known mechanism by which cisplatin exerts its anticancer effects—DNA damage—also affecting renal cells (Tchounwou et al., 2021). Due to the low level of intracellular chloride ions, cisplatin enters the cell and loses its chloride ligand, forming the hydrated form of [Pt (NH_3_)2Cl (OH_2_)] + and [Pt (NH_3_)2(OH_2_)2]2+, which has strong affinity for DNA (Cepeda et al., 2007; Zhu et al., 2015; Russo Krauss et al., 2016). The interaction between the platinum atom in hydrated cisplatin and the purine base’s N7 position in DNA causes the development of intra and interstrand crosslinks. 1,2-strand crosslinks can be formed from adjacent guanine (1,2-GpG) or adjacent adenine and guanine (1,2-ApG), while 1,3-strand crosslinks are formed from non-adjacent guanine (1,3-GpNpG), and interchain crosslinks are formed by guanine. The most frequent adducts produced by cisplatin are guanine-guanine intrastrand crosslinks (1, 2-GpG) (Basu and Krishnamurthy, 2010; Fujikawa et al., 2014). The DNA double helix is generally broken down by these DNA cross-links, which also make DNA replication and transcription more difficult and cause the cell to enter cell cycle arrest mode (Cepeda et al., 2007).

It is interesting to note that cisplatin has been demonstrated to have cytotoxic effects on cells outside of nuclear DNA (Huang et al., 2022). In addition to DNA, Cisplatin can interact with a wide range of biological constituents, including RNA, proteins, membrane phospholipids, and microfilaments that make up the cytoskeleton. Only a small amount of cisplatin interacts with nuclear DNA, whereas a large amount of cisplatin interacts with protein and mitochondrial DNA (Siddik, 2003). A study in Chinese hamster ovary cells demonstrated that the proportion of adducts of cisplatin on mitochondrial DNA was four to six times higher compared to nuclear DNA by dissociation-enhanced lanthanide fluoroimmunoassay (DELFIA) and immunoelectron microscopy (Olivero et al., 1995; Jamieson and Lippard, 1999). This may be because, compared to nuclear DNA, mitochondrial DNA lacks protection from histones and effective DNA repair mechanisms, which makes it more susceptible to damage (Siddik, 2003).

### 3.2 Ischemia-reperfusion injury (IRI) in the kidney

As a highly perfused organ, the kidney is vulnerable to ischemia and reperfusion damage. After reperfusion of blood flow to the ischemic tissues, severe metabolic derangement and tissue structural destruction are observed. IRI is one of the acute renal injuries with a significant mortality and morbidity rate, which mainly manifests as rapid decompensation of renal function (Zhang et al., 2021). It is a complicated pathophysiological process caused by several things that interfere with blood circulation, such as surgery on the urinary and cardiovascular systems, organ transplantation, trauma, shock, and thrombus (Thapa et al., 2022).

Acute renal failure is frequently accompanied by necrosis and malfunction of the tubules, which is caused by ischemia-reperfusion events that harm the polarized tubular cell layer that covers the tubule basement membrane. Tubules may, however, heal themselves by producing new proximal tubular cells to replace damaged or dead cells (Smith et al., 2006). When Rhabdomyolysis occurs, extremely nephrotoxic myoglobin is released into the bloodstream. Myoglobin is filtered by the glomeruli and reabsorbed from the ultrafiltrate by the proximal tubule’s endo cytogenic receptors, giant and cubic proteins, resulting in epithelial cell death (Gburek et al., 2003).

ROS produced during the ischemia-reperfusion (I/R) process in many organs, including the brain, heart, and kidney, induce cellular damage that leads to tissue injury following ischemia. DNA strand breakage, different base or sugar changes, and free nucleotides can all be caused by ROS (Tsuruya et al., 2003). Previous studies have shown that apoptosis, inflammation, angiogenesis, and ferroptosis are all important mechanisms involved in IRI in the kidney, recent studies have also shown that IRI in the kidney is associated with DNA damage and DDR caused by ROS (Yan et al., 2016).

DNA damage occurs in renal tissues after I/R but its mechanisms need to be further investigated. One of the most widely studied is oxidative DNA damage involving 8-oxo-dG (Tsuruya et al., 2003). 8-oxo-dG, the oxidized form of guanine, is cleaved by 8-oxoguanine glycosylase (also known as OGG1). Deletion of OGG1 gene or inhibition of 8-oxoguanine glycosylase results in increased 8-oxo-dG in the DNA and nucleotide pools. It is reasonably stable and will probably build up in genomic DNA throughout typical aging. The accumulation of 8-oxo-dG in DNA increases the incidence of A: T to C: G or G: C to T: A transversion mutations due to the addition of 8-oxo-dG from the nucleotide pool or direct oxidation of DNA (Taddei et al., 1997; Tsuruya et al., 2003).

It has been shown that nitric oxide contributes to renal tubular damage initiated by hypoxia and ischemia. Reactive nitric oxide is known to join superoxide ions to create peroxynitrite, and it has also been demonstrated that the kidney produces peroxynitrite after I/R. Peroxynitrite is also thought to attack DNA directly or indirectly and produce 8-nitro-dG, 8-oxo-dG, and cause DNA double-strand breaks (DSB) (Liu et al., 1996; Tsuruya et al., 2003). Therefore, peroxynitrite is most likely to blame for the buildup of 8-oxo-dG in the renal tubules following I/R damage.

The human OGG1 gene encodes several distinct transcripts as a result of alternative splicing, several of which produce the OGG1 protein in its nuclear and mitochondrial forms. Despite the lack of experimental proof that rodent cells have such nuclear and mitochondrial versions of OGG1, a significant increase of 8-oxo-dG in mitochondrial DNA compared to nuclear DNA has been reported in the liver of OGG1-deficient mutant mice, therefore indicating that OGG1 plays a significant role in the recovery of oxidative damage to mitochondrial DNA (de Souza-Pinto et al., 2001).

DNA methylation can cause DNA damage. Although DNA methylation was the first epigenetic marker to be discovered, research on DNA methylation in AKI is still in its early stages. Renal tubular epithelial cells rapidly produce hypoxia-inducing factor 1 (HIF-1), which results in an epigenetic modification, three to 7 days after kidney ischemia-reperfusion injury (Shu et al., 2019). Genome-wide DNA methylation analysis is necessary for additional mouse models of ischemic and cisplatin-induced AKI, in addition to DNA methylation status study in individuals with AKI. Deep sequencing and microarray-based new methods will enable single-nucleotide and even single-cell precision quantitative study of DNA methylation sites across the genome. The use of these fresh methods will enable us to comprehend DNA methylation’s function in AKI better.

### 3.3 Sepsis-induced AKI

Sepsis is a widespread illness that is, linked to an unacceptably high fatality rate and, for many survivors, long-term morbidity. The modern definition of sepsis is organ dysfunction due to infection (Cecconi et al., 2018). Numerous cell types, including renal tubular epithelial cells, are involved in the pathogenesis of sepsis-AKI (Li et al., 2021). The transfer of oxygen to renal cells is hampered by renal tissue edema, which also worsens local tissue hypoxia. The renal TECs are attacked by the invading inflammatory cells and a high number of inflammatory substances, which results in a decline in renal function 55. Increased levels of ROS are a result of sepsis, which is partly derived from the ferocious immune response that makes it easier to destroy bacteria. In turn, ROS can oxidize proteins, lipids, and DNA, which can lead to collateral damage (Martins et al., 2003; Exline and Crouser, 2008; Angus and van der Poll, 2013). Recently, Nicole Schupp et al. have demonstrated that nuclear and mitochondrial DNA oxidation and damage are mediated by sepsis, particularly a high oxidation of mtDNA, mtDNA damage, and fewer mitochondria in the kidney (van der Slikke et al., 2021).

In kidney illness, ROS comes from two main sources: 1) free radicals that damage the kidney initially, both intracellular and extracellular; and 2) free radicals produced during the inflammatory response caused by the damage (Tucker et al., 2015). Inflammatory cells including neutrophils, eosinophils, and macrophages are infiltration into the injured kidney tissues. The oxidant-producing enzymes, such as NADPH oxidase, myeloperoxidase, and inducible nitric oxide synthase, resulting in large concentrations of various reactive oxygen and nitrogen species (Son et al., 2008). In addition to serving as a means of cellular defense, the ROS that phagocytes release continues to encourage kidney injury or serve as messenger molecules, causing a persistent local inflammatory reaction. Chronic inflammation and recurrent oxidative stress eventually cause glomerular degeneration, which causes serious kidney damage that can be detected, for instance, by a decline in the glomerular filtration rate (Tucker et al., 2015). Normal kidney function is mostly preserved by post-mitotic resting cells in the absence of damage. Renal tubular and interstitial cells can multiply in response to acute or ongoing injury, resulting in tissue remodeling or regeneration (Thomasova and Anders, 2015). Tubular catastrophe and eventual tubular atrophy can result from damaged DNA in renal tubular cells (DiRocco et al., 2014).

### 3.4 AKI-to-CKD transition

At first, clinicians thought that the kidneys possessed a robust ability to regenerate and repair the damage, however, research conducted over the past 20 years has shown that AKI frequently results in partial or even irreversible kidney damage. Interstitial fibrosis, a CKD, develops when structural damage to the kidney continues even after serum creatinine levels return to normal. Renal function recovery relies on adaptive healing in cases of moderate or transient renal injury, where the proliferation of unharmed tubular epithelial cells is predominant. The injury will result in a poor adaptive repair, including aseptic inflammation and fibrosis if it is extensive or chronic (Guzzi et al., 2019). Hypoxia exacerbates hypoxia after kidney damage, which causes renal inflammation and fibrosis, decreased peritubular capillaries, decreased oxygen transport efficiency, increased apoptosis of renal tubular epithelial cells, and ultimately CKD (Guo et al., 2019). Three to 7 days after renal ischemia-reperfusion damage, renal tubular epithelial cells quickly express hypoxia-inducing factor 1 (HIF-1), causing ensuing epigenetic modification (Shu et al., 2019). HIF-1 is made up of *α* and *β* units, which work with anoxic response elements in the regulation area of target genes to control how those genes are expressed when there is anoxia. The “hypoxia memory”-epigenetic alterations derived from hypoxia-can be long-lasting in cells and have an impact on their viability (Li and Li, 2021).

Because of the incomplete knowledge of the mechanisms behind the change from AKI to CKD, there is a lack of therapeutic approaches to directly promote kidney repair or prevent the development of chronic fibrosis. Even though the pathophysiology of AKI is complex, recent investigations have revealed that DNA damage especially DNA methylation in proximal tubular epithelial cells plays an important part in the progression of AKI to CKD (Yang et al., 2010; Ferenbach and Bonventre, 2015; Canaud et al., 2019; Kishi et al., 2019). Clinical research has demonstrated that the etiology of post-AKI CKD involves hypermethylation of genes like Klotho, erythropoietin, and Ras GTPaseactivating-like protein 1 (RASAL1). By lowering the methylation level and increasing the production of these genes, DNMTS inhibitors like 5-azocytosine, 5-azocytosine 2-deoxycytosine, and the antihypertensive hydralazine postpone the progression from AKI to CKD (Chang et al., 2016; Chen et al., 2016; Tampe et al., 2017). Yan Jia et al. have demonstrated that after AKI, kidney fibrosis prevention or treatment with nicotinamide mononucleotide (NMN, an NAD+ precursor) may be an option by considerably reducing inflammation, senescence, and DNA damage in tubular cells (Jia et al., 2021).

### 3.5 Chronic kidney disease

More than 200 million individuals worldwide suffer from CKD, which is a significant public health problem (Irazabal et al., 2022), and studies have shown an increased incidence of cancer in patients with CKD who are on dialysis or transplantation for kidney replacement therapy (Schupp et al., 2016). In the 1990s, there was a study that first suggested that DNA damage exists in patients with CKD (Maisonneuve et al., 1999). After that, more and more studies have shown that DNA damage occurs in conjunction with CKD. Most DNA damage in CKD patients is oxidative, and it mostly takes the form of intra or interstrand crosslinks, crosslinks between DNA bases and proteins, single-strand breaks, double-strand breaks, and oxidized DNA bases (Cadet et al., 2012; Dizdaroglu and Jaruga, 2012).

In CKD patients, the first two indications of genomic damage are sister chromatid exchanges (SCEs) and micronuclei. SCEs are symmetric exchanges of duplicated DNA. Whole chromosomes or chromosomal fragments give rise to micronuclei, the latter arising from unrepaired DNA double-strand breaks that do not reach the spindle poles during mitosis. Since peripheral blood lymphocytes (PBLs) are best suited to study the individual degree of burden of genomic damage, it has been shown that micronuclei are significantly increased in PBLs in CKD patients (Rangel-Lopez et al., 2013). Additionally, there is proof that in PBLs in CKD patients receiving hemodialysis, the number of micronuclei declines (Hoenich et al., 2010).

To evaluate structural DNA damage, single-cell electrophoresis, often known as the comet assay, is frequently employed. According to certain research, peritoneal dialysis patients showed excessive oxidative stress and increased oxidative DNA damage compared to control or non-dialysis patients with CKD (Oberacker et al., 2022). There are also data on non-dialyzed patients with chronic renal failure (stage 4–5) showing that, in agreement with the results of the micronucleus test, a significant increase in comet formation was observed in PBL as well as in salivary gland tissue (Aykanat et al., 2011; Ersson et al., 2011).

The study showed that consistent with data from the comet assay, dialysis patients had increased 8-oxo-dG compared to the normal population. Previous studies have suggested that mitochondrial DNA is more susceptible to oxidative stress, and the findings of this investigation indicate that, technically mitochondrial DNA in dialysis patients double the amount of 8-oxo-dG molecules found in nuclear DNA, but that the relative increase in 8-oxo-dG is greater in nuclear compared to mitochondrial DNA (Kang and Hamasaki, 2002).

As we discussed in the segment on the transition from AKI to CKD, kidney injury factors stimulate the production of the HIF-α in renal tubular epithelial cells, which supports DNA methylation and contributes to renal fibrosis. It has been demonstrated that 5-aza-2-deoxycytidine can reduce the production of fibrotic proteins and kidney fibrosis by removing UUO-induced Klotho inhibition and promoter hypermethylation (Yin et al., 2017).

## 4 DNA repair in renal tubular epithelial cells

In eukaryotes, histone proteins serve as “packing” agents for DNA (Uckelmann and Sixma, 2017; Gong and Miller, 2019). The impacts of DNA damage might affect the superstructures. Damage to DNA mostly affects the double helix’s essential structure. There are several checkpoints built into the cell cycle to make sure the cell is ready to move on to mitosis. The G1/S, G2/M, and spindle assembly checkpoints are the three primary checkpoints that control the passage through anaphase. The G1 and G2 checkpoints are responsible for detecting the presence of DNA damage, with the S phase being the stage of the cell cycle where DNA damage is most likely to occur. At the G2 checkpoint, DNA replication completion and the presence of DNA damage are both examined (O'Hagan et al., 2008).

As we previously stated, environmental influences as well as regular metabolic processes can damage DNA in human cells. These lesions can result in structural damage to the DNA molecule, potentially leading to harmful mutations in the cell’s genome that can affect the survival of daughter cells following mitosis. The DNA repair mechanism is always active in response to damage to the DNA structure. DNA repair refers to the biochemical and molecular biological procedures for removing DNA damage and restoring genomic integrity (Huang and Zhou, 2021). These procedures include DNA damage sensing and signaling, recruitment of repair machinery proteins to the sites of damage, and step-by-step release of these proteins to restore the integrity of the genome. The study of DNA damage repair has quickly branched out into fields including cancer biology, photobiology, and radiobiology. Tomas Lindahl, Paul Modrich, and Aziz Sancar received the 2015 Nobel Prize in Chemistry for their research on the molecular principles underlying DNA repair procedures.

Cells can recognize DNA damage because it changes the spatial organization of the double helix. As soon as the damage is located, specific DNA repair molecules bind to it or the area around it, allowing other molecules to bind and form complexes that allow the actual repair to occur. DNA repair involves numerous standard processes as follows (Figure 1).

### 4.1 Direct reversal

DNA damage can be repaired by cells using chemical reversals. This direct reversal method does not entail a break in the phosphodiester backbone and is specific to the type of DNA damage. Although there is no need for a template for this repair, the kind of damage that is fixed can only happen in one of the four bases. In bacteria, fungi, and most animals, the light reactivation process catalyzed by photolysis enzyme reverses this damage. In human cells, nucleotide excision repairs damage caused by ultraviolet rays. Pyrimidine dimer formation under UV irradiation results in abnormal covalent bonds formed between adjacent pyrimidine bases (Lucas-Lledo and Lynch, 2009). The protein methylguanine methyltransferase can directly undo another sort of damage, guanine-based methylation (MGMT). The third type of DNA damage that cells can repair is specific methylation of the nucleotide’s cytosine and adenine.

### 4.2 Single-strand damage

The other strand of a double helix can be utilized as a template to direct the repair of the damaged strand when just one of the two strands is flawed. To repair damage to one of the two paired molecules of DNA, several excision repair methods remove the damaged nucleotide and replace it with an undamaged nucleotide that is complementary to the undamaged DNA strand.

### 4.3 Base excision repair (BER)

Most frequently, single base or nucleotide damage is fixed by deleting the offending base or nucleotide and replacing it with the proper base or nucleotide. During base excision repair, a glycosylase enzyme eliminates the damaged base from the DNA by cleaving the bond between the base and the deoxyribose. These enzymes produce an apurinic or apyrimidinic site by removing a single base (AP site). At the AP location, AP endonucleases snip the DNA’s broken backbone. After using its 5′to 3′exonuclease activity to remove the damaged area, the complementary strand serves as a template for the DNA polymerase as it correctly synthesizes the new strand. The DNA ligase enzyme then closes the gap (Zhao et al., 2021). A 2015 study found that the degree of kidney fibrosis was positively connected with higher MUTYH expression, a DNA repair enzyme that starts a BER by identifying and deleting 8-Oxoguanine (8-oxoG) and its matched adenine (Lu et al., 2015).

### 4.4 Nucleotide excision repair (NER)

NER is typically utilized to fix bulky, helix-distorting damage initiated by UV light, such as pyrimidine dimerization. Nearly all eukaryotic and prokaryotic cells use NER, a highly evolutionarily conserved repair process. Uv proteins in prokaryotes mediate NER. Many additional proteins are involved in eukaryotes. The DNA polymerase uses the complementary strand as a template to properly create the new strand (Reardon and Sancar, 2006).

According to a study that compared the stress responses of rat kidney tubular (NRK-52E) and glomerular (RGE) cells after exposure to cisplatin, RGE performed the best in repairing Pt-(GpG) intrastrand crosslinks, as evidenced by the high mRNA expression of NER factors (Kruger et al., 2015).

### 4.5 Mismatch repair (MMR)

Almost all cells include mismatch repair mechanisms to fix faults that are not caught by proofreading. At least two proteins make up these systems. The first recognizes the mismatch, and the second calls in an endonuclease to break the freshly manufactured DNA strand adjacent to the area that has been damaged. Following this, an exonuclease removes the damaged area, followed by DNA polymerase resynthesis and DNA ligase nick sealing.

### 4.6 Double-strand breaks

The most serious and fatal form of DNA damage to cells is DNA DSBs. Histone proteins can shield a single strand of damaged DNA from additional damage and chemical attack. The end of the DNA is exposed as a result of DNA DSBs. The intracellular DDR system will become active if this scenario persists without prompt treatment. One of the effects is to halt cell growth and division or to start apoptosis, inducing the cell’s eventual demise. Fortunately, cells have evolved several defenses against DNA DSBs breaks since the eukaryotic stage (Liang et al., 2005).

### 4.7 Homologous recombination (HR)

The cell can access and duplicate intact DNA sequence information in trans through homologous recombination, which is particularly useful for repairing DNA damage that affects the two strands of the double helix. It uses the corresponding feature of the chromosomes in cells. If one of the double-stranded DNA on a chromosome breaks, the other corresponding DNA sequences on a chromosome can serve as a fixed template to respond before breaking the sequence, which is why under certain circumstances homologous recombination is also known as gene conversion.

Cell cycle progression is crucial for the homologous recombination HR repair process. Homologous chromosomes are the sole template that HR can employ in the G1 phase when there are 2n chromosomal sets. The number of chromosomal pairs doubles to 4n with the insertion of a sister chromatid in the S/G2 phase, giving the HR mechanism extra repair templates to choose from. It is widely accepted that HR maintenance operations are more active during the S/G2 period.

### 4.8 Non-homologous end joining (NHEJ)

The most significant difference between NHEJ and HR is that the repair proteins in NHEJ can directly bring the broken ends closer to each other without the help of any template, and then re-join the broken ends with the help of DNA ligase. The mechanism of NHEJ is both simple and templating-independent. In organisms with more complex gene bodies and more junk DNA, NHEJ is more active than HR. However, in organisms with simpler gene bodies, especially single-cell organisms, NHEJ is more likely to break the original sequence integrity.

Double-strand breaks and DNA cross-linkages may result in permanent DNA damage when typical repair mechanisms fall short and cellular death does not take place. The type of cell, the cell’s age, and the extracellular environment are only a few of the variables that affect how quickly DNA is repaired. For a cell that has considerable DNA damage or is no longer able to repair DNA damage correctly, one of three scenarios is possible: Senescence, an irreversible state of dormancy, apoptosis, or programmed cell death, and uncontrolled cell proliferation, which can result in the growth of a malignant tumor. These are three common consequences of senescence. To maintain the integrity of its genome and, by extension, the normal functioning of the organism, a cell’s ability to repair damaged DNA is essential. Many genes that were first thought to affect lifespan have since been found to play a part in DNA damage repair and defense.

## 5 DDR in renal tubular epithelial cells

Some of the processes discussed above can function on their own to repair simple lesions. The DNA damage response, however, controls the repair of more complicated lesions requiring numerous DNA processing steps. The DDR may be crucial for a successful restoration of the lesions that are the hardest to treat. When different types of DNA lesions activate DNA-damage response proteins, DNA repair begins. By phosphorylating repair proteins to change their activities, by initiating a complex chain of modifications to the local chromatin structure close to the damage site, and by altering the cellular environment generally to make it more conducive to repair, these kinases improve the effectiveness of DNA repair (Sirbu and Cortez, 2013). The signaling pathways that these proteins then activate include those for DNA repair, cell cycle arrest, apoptosis, and senescence. In the context of chromatin close to the damage site, complex repair mechanisms take place. Cancers result from dysregulation of DNA damage response and repair, and several of these proteins offer potential therapeutic targets (Figure 1).

The phosphatidylinositol 3-kinase-related kinases (PIKK) oversee the DDR kinase signaling cascades, ataxia telangiectasia-mutated (ATM), DNA-dependent protein kinase (DNA-PKcs), and ATM and Rad3-related (ATR) are included. ATR reacts to a variety of DNA lesions, including those related to DNA replication, while DNA-PKcs and ATM are largely engaged in DSB repair (Cimprich and Cortez, 2008). Because of its adaptability, ATR is crucial for the survival of reproducing cells in both mice and humans (Brown and Baltimore, 2000; de Klein et al., 2000; Cortez et al., 2001).

Three levels of DNA repair are regulated by DDR kinases. First, they directly control DNA repair enzymes’ activity through post-translational changes. When complicated lesions are repaired and when replication forks are halted, these alterations seem to be especially crucial. Second, DDR kinases modify the chromatin around DNA damage to create an environment that is conducive to repair. Additionally, this chromatin response serves as a scaffold to draw in other DDR components that control signaling and repair. Finally, DDR kinases work on the nucleus or possibly the entire cell, creating a biological milieu favorable to repair. This overall response involves alterations in transcription, cell cycle, chromosomal mobility, and deoxynucleotide (dNTP) levels. When damage is persistent, control of these mechanisms might be particularly crucial for repair (Sirbu and Cortez, 2013).

DDR has been the subject of further research lately, including studies on its function in renal tubular epithelial cells. Cellular communication factor 2 (CCN2, also known as CTGF) is a significant factor in the development of CKD and was found to exacerbate DNA damage and the ensuing DDR-cellular senescence-fibrosis sequence after renal IRI. By lowering DNA damage caused by oxidative stress and the ensuing DDR, CCN2 inhibition may lessen the risk of AKI (Valentijn et al., 2021). According to M. Uehara et al., ATM inhibition worsens tubular injury *via* upregulating p53-dependent pro-apoptotic signaling and does not enhance DNA repair following cisplatin-induced DNA damage. When ATM inhibitors are made available in clinical practice in the future, acute renal injury must be closely monitored (Uehara et al., 2020). After kidney epithelial cell damage, Kishi et al. show that DNA repair—rather than cell proliferation—plays a key role in recovery and lifespan by lowering apoptosis, G2/M cell-cycle arrest, and subsequent fibrosis (Molitoris, 2019). By blocking the DNA damage response, p53, MAP kinases, and oxidative/nitrosative stress pathways, JQ1 which is one of the most characterized BET inhibitors with high specificity against BET proteins may protect against the nephrotoxic effects of cisplatin, according to Sun et al. (2018). According to a different study, lovastatin effectively blocks the pro-apoptotic signal pathways of the DDR of injured tubular epithelial cells caused by cisplatin (Krüger et al., 2016).

An essential step in the direct religation of the DSB by NHEJ is the autophosphorylation of DNA-PKcs. The substrates involved in DNA repair, checkpoint signaling, and cell fate decisions including apoptosis and senescence are both distinct and shared by ATM and ATR. PLK3 (polo-like kinase 3) is implicated in oxidative stress-induced DNA damage and TEC apoptosis in renal I/R injury, and PLK3 suppression reduces TEC death after I/R injury by obstructing the ATM/P53-mediated DDR, according to research on the enzyme’s function in renal I/R injury (Deng et al., 2022). Another study found that decreasing the expression of the ATM gene in HK2 cells reduced the amount of LPS-induced inflammation and autophagy in sepsis-induced AKI *in vitro*. This data suggests that the ATM pathway may be used by LPS to cause autophagy in HK2 cells, which would boost the production of inflammatory markers (Zheng et al., 2019). The development of AKI may be influenced by cytoplasmic DNA-PKcs and phosphorylated Fis1 (Wang et al., 2022). For the past several years, our team has been researching the function and mechanism of chronic kidney disease.

## 6 DNA damage and aging

Senescence is a crucial biological process that helps to support embryo development, avoid the growth of tumors, and minimize tissue harm. Senescent cells do, however, play a role in the emergence of age-related illnesses as they accumulate in organs with aging (Shmulevich and Krizhanovsky, 2021). Aging is caused by a variety of variables, including the time-dependent buildup of macromolecular harm, which includes DNA damage and incomplete DNA repair (Yousefzadeh et al., 2021). To escape replicating the damaged genome, sustained DNA damage (genotoxic stress) sets off a cascade of signals that cause apoptosis or senescence. At the same time, these processes encourage cellular senescence (Babbar et al., 2020; Yousefzadeh et al., 2021). A growing body of data points to inflammation as another significant side effect of DNA harm. One of the signs of aging and the primary cause of many age-related illnesses is inflammation (Zhao et al., 2023). The buildup of age-related DNA damage, activation of transposons, cellular senescence, and accumulation of persistent R-loops are thought to be the triggers of these signaling cascades, which are activated by engaging the cGAS-STING axis or activating NF-kappaB *via* ATM. Meanwhile, the shift of heterochromatin components mediated by epigenetic modifications generated by DNA harm can result in inflammation and aging (Zhao et al., 2023). AKI and the aging activation pathway are closely linked. Tubular cells suffer damage as a result of kidney toxicity or ischemia-reperfusion injury, which is primarily expressed as cell membrane damage, cytoskeleton damage, and DNA degradation. These damages eventually cause tubular cell necrosis, apoptosis, and mortality (Andrade et al., 2018). Age-related processes like interstitial fibrosis, renal tubule atrophy, and scant capillaries make it difficult for the kidneys to recoup from structural and functional damage, which raises the possibility that AKI and the development of chronic kidney disease are closely related (Andrade et al., 2018; Kim et al., 2021). Studies in recent years have also concentrated on the connection between chronic renal illnesses like diabetic nephropathy and cell aging. Diabetic nephropathy is caused and developed by DNA damage, epigenetic change, and mitochondrial malfunction (Xiong and Zhou, 2019).

## 7 Gender differences in DNA damage and repair

Clinical epidemiological studies have shown that while CKD is more common in women than in men, end-stage renal disease, including dialysis and kidney transplantation, is more common in men (Carrero et al., 2018). There is mounting evidence that the way each gender responds to kidney injury differs significantly. The tolerance for renal ischemia-related impairment varies by gender. Although recent research suggests that male hormones also play a significant role in these differences, it has long been believed that the protective effects of estrogen account for sex differences in disease susceptibility. Gender and sex hormones appear to have an impact on vascular factors like endothelin, nitric oxide, and angiotensin II (Metcalfe and Meldrum, 2006). Additionally, inflammatory mediators with different expression and activity based on sex and the presence of sex steroids included TGF-β1, TNF-α, and p38 mitogen-activated protein kinase. Studies have revealed that the renal toxicity of cisplatin varies significantly by gender (Pezeshki et al., 2017; Valdivielso et al., 2019). Proteomic research on the disruption of dihydrotestosterone metabolism in human primary proximal tubule epithelial cells raises the possibility that changed tubular energy metabolism may be connected to the harmful effects of androgens on the kidney (Valdivielso et al., 2019).

There are also gender differences in DNA damage and repair especially DSB, according to a growing body of studies. One of the studies has shown that the chance of developing Alzheimer’s disease (AD) may be influenced by the presence of more DSB markers in the hippocampus of female E4 mice (Boutros et al., 2023). Males and females experience different age-related changes in DSB repair, according to research that examined DSB repair in peripheral blood lymphocyte (PBL) circulation in male and female donors of various ages (Rall-Scharpf et al., 2021). According to one research, the new tank tests had a greater impact on female zebrafish, who displayed constrained locomotion and exploratory behavior, decreased mitochondrial enzyme activity, increased DNA damage, and cell death after hypoxia damage (Das et al., 2019). The effect of the aromatic amine 2-acetylaminofluorene (AAF) on hepatocellular carcinoma induction in male rats was more pronounced than in female rats, demonstrating that the AAF-DNA adduct in male rats was approximately twice as large as that in female rats, and the DNA repair level in male rats cultured with AAF dosage was approximately three times greater than that in female rats (Williams et al., 2016).

The question of whether DNA amplification, DNA repair, and DDR are gender-related in kidney disease has received very little attention in studies and articles up to this point. It is beneficial for clinicians and researchers to have a deeper understanding of the specific role of DNA damage in kidney disease as it may provide a more accurate treatment direction for the prevention of kidney disease and kidney injury in the future. This will help to improve the understanding of sex and sex-specific differences in the etiology, mechanism, and epidemiology of kidney disease, as well as to combine the factors related to DNA damage and sex.

## 8 Interventions targeted DNA damage in renal diseases

As discussed earlier, DNA damage is a common occurrence in a variety of renal diseases. Numerous studies have focused on DNA damage in kidney disorders, including treatments that target DNA damage specifically. According to one study, miR-155 deficiency dramatically lessens pathology and mortality in cisplatin-induced AKI by preventing DNA damage (Yin et al., 2022). Another study has demonstrated that when compared to normoxia-cultured mesenchymal stem cells, hypoxic mesenchymal stem cells (HMSCs) demonstrated a superior anti-oxidative impact in I/R-damaged rat kidneys *via* reducing DNA damage (Tseng et al., 2021). A study on the prevention of chronic fibrosis revealed that NMN could drastically reduce DNA damage, senescence, and inflammation in tubular cells. As a result, giving NMN may be an effective way to stop or cure renal fibrosis after AKI (Jia et al., 2021). Additionally, M. A. Mohammed et al. have demonstrated that vitamin D protects against oxidative stress and DNA damage to lessen the acute renal damage caused by gentamicin (Mohammed et al., 2019). Therefore, focused approaches to DNA damage may be effective methods for kidney protection in renal diseases.

## 9 Perspective

DNA damage alters the structure of genetic materials, which makes it impossible for the replication mechanism to work properly. In response to the presence of DNA lesions, cells could either be repaired or eliminated by triggering cell death after DNA repair fails. When DNA is damaged, DNA repair proteins are frequently activated or induced. However, if the level of DNA damage is too severe to repair, the organism has acquired the further option of initiating an apoptosis program that can stop excessively damaged cells from mutating and developing into cancers. The complicated signal transduction network known as DDR detects when DNA is damaged and starts the cellular response to the damage. While most of the DNA damage can be repaired, such a repair function is not 100% successful all the time.

Since DNA damage has been implicated in a variety of kidney injuries, its early and sensitive detection in kidney disease by specific assays may become a new target for early diagnosis or prognosis of the disease. In addition, targeting DNA damage and DNA damage response may well be a successful kidney protection technique in AKI treatment.
